# The association between sleeping behavior, obesity, psychological depression, and eating habits among adolescents in the emirate of Abu Dhabi–United Arab Emirates

**DOI:** 10.1371/journal.pone.0269837

**Published:** 2022-08-30

**Authors:** Rania Al Dweik, Yousef Sheble, Hiba Ramadan, Haneen Issa, Abdullah Sheble

**Affiliations:** 1 College of Health Sciences, Abu Dhabi University, Abu Dhabi, United Arab Emirates; 2 School of Dentistry, Queen’s University Belfast, Belfast, United Kingdom; 3 School of Medicine, UCLAN University, Lancashire, United Kingdom; University Sains Malaysia, MALAYSIA

## Abstract

**Objective:**

The study aimed to investigate the association between sleeping behavior (specifically sleep duration), body mass index (BMI), eating habits, and psychological mood depression among adolescents in the Emirate of Abu Dhabi- UAE.

**Methods and materials:**

A subsample of three hundred and ninety-five participants (209 females and 186 males) from middle and high schools (aged 12–18 years) in the emirate of Abu Dhabi completed the surveys in the presence of their parents and two research assistants. Measures of daytime sleepiness and other sleep parameters (sleep duration on weekdays and weekends), eating habits, and mood depression questionnaires were reported.

**Results:**

Differences in BMI between males and females were statistically significant (26.12 ± 4.5 vs. 24.4 ± 4.3; *p* < 0.01). There was a negative linear association (*p* < 0.01) between the students’ BMI and the weekday/ weekend sleep duration. The average weekday and weekend sleep duration ranged from 5.7 hours (weekdays) to 9.3 hours(weekend). The study showed that an increase in BMI was correlated to mood depression (r = 0.396, *p*<0.01). In terms of eating habits, there was a significant association between eating unhealthy food and sleep duration; 72.6% of students who slept less than 6 hours reported unhealthy eating habits (p <0.05).

**Conclusion:**

The study showed a clear association between short sleep duration and obesity among adolescents in the UAE. This relationship between sleep duration and obesity is less studied and less understandable. Future research about exploring how sleeping behaviors can affect obesity during adolescence can support understanding this association and create an effective intervention.

## 1. Background

The prevalence of overweight and obesity among children and adolescents has increased significantly in several countries, including the UAE in the past few decades [1]. In 2013, a randomized control study that included 1541 students (grades 1–12) in 246 schools in Abu Dhabi, showed that 18.95% were obese, 14.7% overweight, and 7.6% underweight [2]. Obesity can lead to adverse medical conditions in adolescence and adulthood [3–5]. Being overweight often results in a lower quality of life, reduced self-esteem [6, 7], poor performance in school [8], and short sleep duration [9]. Obese individuals are at high risk of developing medical comorbidity as well as social, and emotional complications [10].

New evidence from different cross-sectional studies conducted internationally indicates that shorter sleep duration is a risk factor for higher BMI in children and adolescents, as well as in adults, although the relationship appears to be somewhat stronger in children [11]. This relationship is common when sleep duration is less than six hours per night and the individual falls asleep after midnight [12, 13]. Similar outcomes have been obtained regarding the relations between sleep problems and long-term weight gain [14]. A significant relationship between obesity and short sleep duration was reported in a large meta-analysis of sleep data gathered from children and adults [15].

Another study that included 74,000 students in South Korea found an inverse association between sleep duration and BMI and being overweight or obese [16]. One hour less in sleep duration resulted in an increased likelihood of overweight or obesity by 6.5% [16]. Other studies have reported lifestyle and psychological factors’ effects on obesity, such as sedentary behaviors, inactivity, and poor diet [4, 17].

The highest rates of excess weight among adolescent males and females were found in North America, and southern and eastern European countries [18, 19]. In many countries, males tend to be more obese than females, probably because females eat more healthy foods and males eat more fast foods [20], or because parents do not frequently encourage males to control their weight [21]. Females tend to gain body fat during puberty, which may serve as an incentive for initiating dieting practices [22].

To the best of our knowledge, the relationship between sleep duration, BMI, mood depression, and eating habits has not received much attention, and no study in the UAE has examined this relationship among adolescents 12–18 years of age. This study aimed to examine how self-reported sleep duration is associated with obesity among adolescents in grades 7–12 (aged 12–18 years) in UAE.

## 2. Methods and materials

This observational cross-sectional study was conducted on 395 adolescents (207 females and 188 males) from middle and high schools (aged 12–18 years) in urban and rural middle-class communities in the emirate of Abu Dhabi–United Arab Emirate. All participants were English speakers and studied in grade 7–12 classes. An introduction to the study was shared with the participant’s guardians explaining the aims of the study. The participant’s guardians signed a consent form that was approved by the ethics committee and the questionnaires were completed in the presence of the participant’s guardians and a research assistant. The participants completed demographics, sleep duration, and depression severity questionnaires in the presence of their guardians and two research assistants.

Age, gender, and grade in school were reported by the participants. Research assistants took the participants’ height and weight, which was used to calculate the BMI (BMI expresses the weight/height relationship as a ratio of kilograms/meters2) [23, 24]. As to Cole et al, 2000; normal weight was defined as BMI between 18.5 and 24.9; excess weight as BMI between 25 and 29.9, and obesity as BMI ≥ 30 [25, 26]. The prevalence of obesity among adolescents was measured.

A self-reported questionnaire including sleep problem behaviors, daytime sleepiness, and depressed mood scale was used to assess sleep patterns. This included bedtime (h: min), sleep latency (the time it takes from light off until sleep onset), wake-up time (h: min), and total sleep duration (hours), both on weekends (Friday and Saturday) and weekdays (Sunday to Thursday).

Mood Scale Patient Health Questionnaire (MSPHQ) was used to assess the depression severity score of the participants. The last version of the MSPHQ included 9 items such as “poor appetite or overeating” or “Feeling down, depressed, or hopeless”. The questionnaire was divided into three subscales: mild depression (a score of 5–9), moderate depression (a score of 10–14), and severe depression (a score of 15 or more).

Pearson correlations test was used to find the association between sleep measures and BMI as well as the association between psychological severity of depression and BMI. Statistical analyses were performed using SPSS version 26 statistical software.

The study was approved by the Abu Dhabi University Institute and Research committee. An introduction to the study was shared with the participant’s guardians explaining the aims of the study. Questionnaires were completed in the presence of the participant’s guardians and a research assistant.

## 3. Results

A total of 395 adolescents from middle and high schools were participated in the survey, including 209 females (mean age 14.9 ± 2.08 years, range 12–18; mean BMI 24.4 ± 4.2; range 16.7–40.7) and 186 males (mean age 14.9 ± 2.06 years, range 12–18; mean BMI 26.2 ± 4.5; range 18.2–40.0). Three percent of the questionnaires were partially completed and were therefore removed from the analysis (Table 1).

**Table 1:** Sleeping behavior, mode scale, and BMI comparison between male and female participants.

	Female	Male	Significance
Age (years)	(14.9±2.08)	(14.9 ±2.06)	NS
Sleeping Behavior			
Weekday sleep duration	(5.68±1.5)	(5.76 ±1.5)	NS
Weekend sleep duration	(9.30±1.86)	(9.34±1.80)	NS
Mode scale	15.64 ± 6.30	20.21± 7.24	P<0.01
BMI	24.4 ±4.3	26.2 ± 4.5	P <0.01

The difference between bedtime and wake-up time minus the sleep latency formula was used to compute the sleep duration during both weekdays and weekends. Asleep latency of 0.30 (the median sleep latency) was used for students who reported no weekday sleep latency or whose recorded weekday sleep latency was greater than 1 h (n = 12), and for students who reported no weekend sleep latency or whose recorded weekend sleep latency was greater than 1 h (n = 30). Three variables of sleep duration were used for data analysis: weekday night sleep, weekend night sleep, and mean daily night sleep (average duration of weekday and weekend night sleep). The prevalence of students with insufficient (7 h or less) and long (9 h and more) sleep duration was measured [27].

The prevalence of students with normal BMI (18.5 < BMI < 25) was 231 and among those overweight or obese (BMI ≥ 25) were 164 students. Males reported a statistically significant higher BMI of 26.2 ± 4.5 than females 24.4 ± 4.3 with p < 0.01. The odds of a male adolescent having a BMI >25 were 1.5 times higher than that female (OR:1.49; 95% CI) (Table 2).

**Table 2:** Sleeping behavior and BMI among participants.

	N	Weekday sleeping behavior			Weekend sleeping behavior			Weight	
		Weekday duration (hrs.)	Sleep ≤ 7 hrs.	Sleep >9 hrs.	Weekend sleep duration	Sleep ≤7 hrs.	Sleep >9 hrs.	BMI (N = 395)	BMI > 25
			N (%)	N (%)		N (%)	N (%)		N (%)
Total	395	5.72 ± 1.54 (2.58 – 13.00)	345 (87.34%)	12 (3.03%)	9.32±1.81 (1.5 – 14.0)	41(10.39%)	209 (52.9%)	25.26 ± 4.54 (17.6 -40.7)	173 (43.8%)
Gender									
Male	186	5.75 ± 1.57	165 (47.8%)	7 (58.3%)	9.33 ± 1.80	16 (39.0%)	99 (47.3%)	26.2 ± 4.52	93 (53.7%)
Female	209	5.69 ± 1.59	180 (52.1%)	5 (41.7%)	9.30 ± 1.87	25 (60.9%)	110 (52.6%)	24.4 ± 4.40	80 (46.2%)
School-level									
High School	199	5.72±1.65	174 (50.4%)	9 (75.0%)	9.27 ± 1.89	22 (53.7%)	98 (46.9%)	26.7 ± 5.38	91 (52.6%)
Middle School	196	5.71±1.43	171(49.6%)	3 (23.0%)	9.37 ± 1.79	19 (46.3%)	111 (53.1%)	24.9 ± 4.34	82 (47.4%)

Male and female participants slept an average of 5.7 hrs. on a weekday and 9.3 hrs. on the weekend. There was no statistically significant difference between male and female students in terms of sleeping hours during weekdays (males: 5.77; females: 5.69, p<0.51) and weekends (male:9.3; female:9.2, p<0.88). During weekdays, 72.7% of the students slept 6 hours or less, 8.8% slept about 7 hours, and only 3.3% slept more than 9 hours. During weekends, 5.82% of the students slept less than 6 hours, 3.53% slept about 7 hours, and 73% slept more than 9 hours. Also, there were no statistically significant differences in the number of hours students slept during the weekdays (middle school: 9.4; high school: 9.3 (p<0.58) and weekends (middle school:9.3; high school:9.2 (p<0.58) by the level of education. (p<0.17 and p <0.32 respectively).

The results showed a negative association between the percentage of students with BMI > 25 and weekday sleep duration (p <0.01) for both genders. The higher the BMI the lower duration of sleep. Also, a strong positive relationship was found between the mode scale and BMI > 25 with p (<0.01). The higher the depression score was associated with higher BMI in both males and females.

In terms of unhealthy eating habits, females showed a higher percentage (53.7%) compared with males (46.3%) at a p-value <0.05. Females tend to choose more unhealthy food than males. However, there was no statistically significant difference between males and females in terms of healthy eating habits. Also, an association between unhealthy eating habits and sleep duration was found, where 77.6% of students who slept less than 6 hours reported unhealthy eating habits (p <0.05). There was a significant association between school grading and eating habits. Students at middle schools reported a higher percentage of unhealthy eating habits compared with high school students at a p-value <0.01.

Male participants showed a higher depression severity score (20.2 ±7.2) than females (15.64±6.3) at p (<0.01). Also, there was a negative association between sleep duration and mood depression. The sleep duration was shorter for the participants with higher mood depression scores (Table 3).

**Table 3:** Correlation between BMI, sleep duration, eating habits, and mood.

	BMI		Weekday sleep duration		Weekend sleep duration	
	*r*	*p*	*r*	*p*	*r*	*p*
BMI			-0.144	<0.05	- 0.119	0.097
Eating habits						
Healthy	0.053	0.46	0.100	<0.05	0.058	<0.05
Unhealthy	0.197	< 0.01	-0.59	<0.05	-0.110	<0.01
Mood depression	0.396	< 0.01	- 0.114	<0.05	0.181	<0.01

## 4. Discussion

Obesity is a huge problem among adolescents. The increasing prevalence of adolescent obesity is associated with a rise in comorbidities previously identified in the adult population, such as Type 2 Diabetes Mellitus, Hypertension, Non-alcoholic Fatty Liver disease (NAFLD), Obstructive Sleep Apnea (OSA), and Dyslipidemia [28]. This study was designed to explore the relationship between sleeping behavior, BMI, eating habits, and mood disturbance among UAE adolescents.

A negative association was found between BMI and sleep duration among adolescents. This was confirming the research hypothesis that a short sleeping duration will result in increasing BMI. The UAE adolescent’s average sleeping hours per night (7.5 hrs.) findings were consistent with the previously reported sleeping duration of both the US high-school students (7.8 hrs. per night) and the Israeli adolescent students (8 hrs. per night) [20, 28]. The reported sleeping hours by the UAE adolescents were lower than the recommended by the American Academy of Sleep Medicine for this group (8 to 10 hours per 24 hours) [29]. Also, an association between short sleep duration and an increase in BMI was found. The BMI for the students with short sleep duration was higher than the students with normal sleep duration.

Similarly, Kilani et al, (2013), found that 42.5–57.6% of Omani adolescents do not get enough sleep. The average sleep duration of 6.7 hrs. appears lower than that reported for Saudi Arabian adolescents (Olds, Maher, Blunden, & Matricciani, 2011), adolescents in the US (McKnight-Eily et al., 2012), and the Israeli adolescents which ranged from 6.8, 8.7, and 8 hrs. for 14–19 15.5–17.5, and 12 to 18 –year—old respectively.

In terms of eating habits, females (53.7%) tend to eat more unhealthy diets than males(46.3%) at a p-value <0.05. These results were consistent with Musaiger and Kalam’s (2014) study that indicated a significant variation in eating habits between male and female adolescents in Syria [30]. Also, an association between unhealthy eating habits and sleep duration was found, where 77.6% of participants who slept less than 6 hours reported unhealthy eating habits (p <0.05). This is similar to the finding reported by Simon SL et al 2019, who found that shorter nighttime sleep duration was associated with higher sugar intake [31]. Also, Westerlund, Ray, and Roos (2009) showed that among 10–11-year-old children, shorter sleep duration is associated with the consumption of more energy-rich foods and fewer nutrient-dense ones, with boys exhibiting a stronger association than girls [32].

A positive relationship between sleep duration and mood depression was reported (p<0.05). Mood depression was highest among participants with both sleep disturbance and short sleep. Nguyen-Rodriguez, McClain, and Spruijt-Metz (2010) found that sleep onset latency among 356 adolescent students was significantly related to emotional eating, depressive symptoms, and anxiety traits [33].

According to Owens et al 2014, adolescents who do not get enough sleep have a higher risk of obesity, diabetes, injuries, poor mental health, and problems with attention and behavior [34]. Many correlational studies have found a strong relationship between short sleep, bedtimes after 9 p.m., and obesity risk [35, 36]. Although the exact mechanisms for this association are still not clear, short sleep duration may lead to a greater number of eating opportunities due to increased waking hours [37–39], greater daytime fatigue and (potential) consequential lower energy expenditure, and/or fluctuations in appetite and hunger hormones [40, 41]. Recently, Wheaton et al. (2013) also found that very short sleep duration is associated with an increased prevalence of weight-control behaviors, especially unhealthy ones, in both male and female high school students [42].

Over the last decade, many epidemiological studies reported an association between short sleep duration and obesity in adolescents, this relationship between sleep duration and obesity is less studied and less understandable. Exploring how sleeping behaviors can affect obesity during adolescence can support understanding this association and create an effective intervention.

## 5. Research strengths and limitations

To our knowledge, this is the first UAE study to explore the association between sleep duration, BMI, eating habits, and mood depression among adolescents. One of the limitations of the study was the potential for recall bias in sleep duration and eating habit reporting. Other limitation includes reliance on participants’ subjective reports rather than objective measures. One of the strengths of this study includes recoding the weight and height of the research assistants which interpreted BMI data more accurately.

Future research must examine BMI and sleep behavior using objective tools and actual observations of eating habits, and quality of life. Decreased sleep duration was found to be related to unhealthy eating behaviors. The results provide further evidence that inadequate sleep may contribute to an increase in risky behaviors among adolescents. Because this is the latest study to document an association between weight-control behaviors and short sleep duration in the general adolescent population in the UAE, more studies are warranted to confirm these results.

## 6. Conclusion

In summary, this study explored the direct links between short sleep durations and attitudes and beliefs that are antecedents of obesity. Further intervention studies should be conducted to; explore the effect of educational interventions that target sleep behavior and eating habits, and to highlight the association between using screen-based devices and sleep behavior and eating habits
